# Knowledge and practice of tuberculosis infection control among health professionals in Northwest Ethiopia; 2011

**DOI:** 10.1186/s12913-014-0593-2

**Published:** 2014-11-19

**Authors:** Chanie Temesgen, Meaza Demissie

**Affiliations:** ᅟ, Bahir Dar, Ethiopia; Addis Continental Institute of Public Health, Addis Ababa, Ethiopia

**Keywords:** ᅟ

## Abstract

**Background:**

Tuberculosis (TB) is highly prevalent in sub-Saharan Africa, making the risk of infection transmission high in these countries. Despite high prevalence of TB and expected high probability of nosocomial transmission in Ethiopia, a rapid assessment done in 2008 revealed that most health facilities in Ethiopia do not use tuberculosis infection control (TBIC) practices. Patients and providers are therefore at risk of exposure to TB, especially at high case load facilities. The purpose of this study was to assess TBIC knowledge and practices among health professionals working in hospitals in the Amhara region of Northwest Ethiopia.

**Methods:**

An institution-based hybrid study was implemented form August 2010 to January 2011. The subjects were health professionals who were proportionally selected from each hospital. Subjects self-administered a questionnaire that contained sections on socio-demographics and on TBIC knowledge and practice. Those answering ≥60% of knowledge questions correctly and ≥50% of practice questions correctly were considered to have good knowledge and practice, respectively.

**Results:**

A total of 313 healthcare professionals were enrolled from four healthcare facilities. The response rate was 96%. Only 18.8% received in-service training. Among those who were trained, 74.4%, 95% CI (69.6, 79.3%) were found to have good knowledge and 63.2%, 95% CI (57.9, 68.6%) good practice on TBIC. Training was found to be a predictor of TBIC knowledge, AOR* 3.386 and 95% CI (1.377, 8.330) while knowledge of TBIC was a strong predictor of good TBIC practice, AOR* 10.667 and 95% CI (5.769, 19.721).

**Conclusions:**

Though the majority of the respondents had good TBIC knowledge and practice, a considerable proportion of healthcare professionals were not trained on TBIC. Respondents trained on TBIC were found to be more knowledgeable than those not trained. Similarly, respondents with good TBIC knowledge were 10 times more likely to have good TBIC practice compared to those with poor TBIC knowledge. Training was not found to have an effect on TBIC practice.

*Adjusted Odds Ratio.

**Electronic supplementary material:**

The online version of this article (doi:10.1186/s12913-014-0593-2) contains supplementary material, which is available to authorized users.

## Background

Tuberculosis infection control (TBIC) is a combination of measures aimed at minimizing the risk of TB infection transmission within populations [1]. Gaps in (TBIC) implementation practice predispose health professionals to nosocomial tuberculosis (TB) transmission. TBIC is not appropriately implemented in most developing countries. Effective TBIC in healthcare settings depends on early identification, isolating infected persons, and rapidly and effectively treating persons with TB. A combination of control measures--including administrative, engineering, and environmental controls and personal protection measures--have been recommended to reduce nosocomial TB risk [2]. These recommended measures are implemented by healthcare facilities in high-income countries; however, given their high cost, few facilities in low-income countries can afford to implement them [3].

“The risk of transmission of *M. tuberculosis* from individuals with TB to other patients and to health professionals (HPs) has been recognized for many years. This risk is high in health facilities especially in many low- and middle-income countries” [4]. A review of available published literature regarding the risk of TB infection and disease among health care workers (HCWs) revealed that the median prevalence of latent TB infection (LTBI) in HPs was 63% [5]. Other reports showed tuberculin skin test conversion and increased risk of TB among health professionals in Turkey [6] and Melbourne [7], demonstrating the importance of appropriate TBIC practice at health care settings.

Despite the high prevalence of TB and the expected high probability of nosocomial transmission in Ethiopia, a recent assessment established that most health facilities have do not use TBIC practices [8]. Patients and providers are therefore at risk of exposure to TB, especially at high case load health facilities where TB patients and suspected cases stay longer for inpatient or outpatient follow-up care.

Recommended TBIC measures to reduce nosocomial infection--including developing an infection control plan, educating health professionals and patients, improving sputum collection practices, performing triage and evaluation of suspected TB patients in outpatient settings, reducing exposure in the laboratory [9], and employing administrative controls (early detection, isolation, and treatment of patients with TB)--have been the most effective components of TBIC programs [10]. Delays in diagnosis and initiation of treatment and failure to separate or isolate patients with smear-positive TB from other patients also contribute to transmission risk [11,12].

## Methods

The study involved four hospitals that were located between 300 km (Debre Markos) to 567 kms (Bahir Dar) northwest of Addis Ababa. The study hospitals were selected based on the flow of TB patients. Study hospitals combined are expected to serve a total population of more than 9 million.

This study used a health institution-based hybrid design. The staff from the four study hospitals served as a source population, and 313 of these healthcare workers were enrolled in the study. The four hospitals have a total of 498 health care workers. The group of study participants was composed as follows: 13% from Motta hospital, 13% from Finoteselam hospital, 31% Debre Markos hospital, and 43% from Felegehiwot hospital. The source population was composed of physicians, health officers, nurses, laboratory technicians, pharmacists, radiographers, physiotherapists, and environmental health workers.

A list of all health professionals in each hospital was prepared and used to select 326 study subjects. The sample size was determined by using EPI INFO Version 3.5.1 Ssat calc by considering a 50% level of TBIC practice, a 95% confidence level, 80% power, and a 1:1 ratio of poor and good practice to detect an odds ratio (OR) of 2. A ten percent (10%) non-response rate was considered to calculate the final sample size. Proportional numbers of study participants were allocated to each hospital according to its contribution to the sampling frame and individual participants were selected by a lottery method from each facility, taking the contribution of each discipline into consideration. Study participants were selected from units where TB patients and suspected cases receive health care.

Trained data collectors facilitated the self administered data collection by using a pre-tested structured questionnaire focused on knowledge and practice related to TBIC. With the assistance of coordinators in each unit, data collectors had all study participants in the same department complete the instrument simultaneously. The principal investigator supervised data collection to ensure data quality and implemented a daily check of the collected data in order to keep consistency.

Respondents answering ≥60% of the TBIC knowledge questions correctly were considered to have good TBIC knowledge; others were considered to have poor knowledge. Those answering ≥50% of the TBIC practice questions correctly were considered to have good TBIC practice, and others were considered to have poor practice.

TBIC knowledge (good and poor) and TBIC practice (good and poor) were taken as the main dependent/outcome variables. Explanatory/exposure variables potentially determining TBIC knowledge and practice were the socio-demographic characteristics: year of service, level of education, professional category, job location, and training on TBIC. Knowledge related to TBIC was also considered as a potential explanatory variable for TBIC practice.

Data were cleaned and entered into a computer using EPI-INFO version 3.5.1. Data were then exported and analyzed using SPSS version 15. Variables were recoded to compute some of the analysis. Frequencies, percentages, and means were calculated as appropriate for TBIC knowledge and practice. Percentage compliance of TBIC practice was calculated. The relationships of independent/predictor variables (age, gender, level of education, training, job location, professional category, and service year) with dependent variables (good knowledge, poor knowledge, good practice, and poor practice) were calculated through cross tabulation and a summary table was generated. Univariate binary logistic analysis and multivariate logistic regression analysis were also done to determine the relationship between outcome variables and a range of factors.

Odds ratios (OR) were computed for the presence and strength of the associations found and 95% CIs were calculated to determine statistical significance for each predictor variable. Finally, those variables that showed association were analyzed using multivariate models. Only variables that showed statistically significant association in the final analysis were considered to explain the presence of association.

Ethical clearance was obtained from the Joint MPH program of Addis Continental Institute of Public Health and Gondar University. Officaly written permission was secured from the Amhara Regional Health Bureau. The respondents were informed about the objective and purpose of the study and each respondent gave verbal consent. Respondents were also informed of their right to participate or not participate in the study. Confidentiality of the information was assured; data was collected anonymously and only an identity number was used on each questionnaire.

## Results

A total of 313 healthcare professionals were enrolled from four healthcare facilities. The response rate was 96%. Among the respondents, 153 (48.9%) were males and 160 (51.1%) females. The median age was 28; the mean age was 30.3, and age ranged from 20 to 60 years. Concerning the educational level of the respondents, 198 (63.3%) have a diploma (55.9% were either clinical nurses or midwives) and the remaining 115 (36.7%) had a university degree or post graduate work.

One hundred and eighteen (37.7%) of the respondents work in the outpatient department, 119 (38%) were from the wards, and the remaining 76 (24.3%) were from either the laboratory or pharmacy.

Only 18.8% of the respondents were trained on TBIC. Of these, 45% were trained in the past year while 55% were trained in the past two or more years. For 85% of those trained, the TBIC trainings lasted for a period of three or more days (see Table 1).

**Table 1:** **Socio-Demographic characteristics of respondent health professionals**

**Variables**		**Number**	**Percent**
Age	18- 29	196	62.6
	30–39	67	21.4
	>/ = 40	50	16
Sex	Male	153	48.9
	Female	160	51.1
Educational Background	Diploma	198	63.3
	Degree and above	115	36.7
Category of health workers	Physician or HO	59	18.8
	Nurse	175	55.9
	Lab, pharm, or other	79	25.2
Job location	OPD	118	37.7
	Wards	119	38
	Lab or Pharm	76	24.3
Years of Service	<10	236	75.4
	10-19	43	13.7
	>/ = 20	34	10.9
Training on TBIC	No	254	81.2
	Yes	59	18.8

Key: HO = Health Officer, Lab = Laboratory professional, Pharm = Pharmacy professional, OPD = Out Patient Department.

### Tuberculosis infection control knowledge and determinants

When assessing TBIC knowledge, 78.3% of the respondents mentioned the need for a TBIC committee. On questions asking them to identify effective TBIC measures, 88.5% identified opening a window; 77.3%, isolation; and 64.5%, minimizing hospital stays. Respondents identified other TBIC measures as well: 34.2% identified use of a respirator by the health worker; 86.3%, educating patients; 75.7%, prioritizing TB suspects; and 71.6% health worker TB screening. Overall, 74.4% of the respondents were found to have good knowledge. On the other hand, only 34.2% of the respondents knew that respirators can provide protection from inhaling *mycobacterium tuberculosis* (MTB) *bacilli* and only 46% correctly identified that use of a fan (ventilator) minimizes the risk of TB infection.

Multivariate logistic regression models that included variables for training on TB, job location, and age category revealed that training is the strongest determinant of knowledge, AOR 3.386 and 95% CI (1.377, 8.330). On the other hand, job location and age category, AOR 0.592 and 95% CI (0.286, 1.223) and 0.913 95% CI (0.649, 1.284), respectively, were not found to be associated with TBIC knowledge in the multivariate models (Table 2). Neither did years of service or education levels show any association with TBIC knowledge.

**Table 2:** **Multivariate logistic regression of TBIC knowledge using predictor factors identified in univariate logistic regression**

**Characteristics**	**Poor knowledge**	**Good knowledge**	**COR 95% CI**	**AOR 95% CI**
Age Category				
30-39	25	42	0.486 (0.267, 0.884)*	0.913 (0.649, 1.284)
18-29	44	152	1	1
Job location				
Laboratory and Pharmacy	14	62	2.104 (1.048,4.222)*	0.592 (0.286,1.223
OPD	38	80	1	1
TBIC training				
Yes	6	53	3.613 (1.496, 8.813)*	3.386 (1.377, 8.330)*
No	74	180	1	1

Note: *Significant association.

### Tuberculosis infection control practices and determinants

Questions concerning the health professionals’ TBIC practice revealed that 64.9% (*n =* 203) of the respondents do open windows before they start work to improve the natural ventilation of the room. In addition, 71.6% of them educate patients on how to prevent TB infection transmission and 60.7% replied that they get tested for TB when they have symptoms of the disease. Only 21.1% of healthcare workers indicated that they use a mask to protect themselves from TB infection. Finally, 33.5% said they give priority to TB patients, and 39.9% use a fan (ventilator) to augment the natural ventilation. Overall, 198 out of 313 health professionals (63.3%) had “good” scores for practice regarding TBIC. The analyses found no significant difference in the proportions of male and female respondents with good TBIC practice (64.1% and 62.5%, respectively), OR 0.935 and 95% CI (0.591, 1.482).

Multivariate analysis (job location and TBIC knowledge) revealed that knowledge about TBIC was the strong predictor of good TBIC practice, AOR 10.667 and 95% CI (5.769, 19.721) (Table 3).

**Table 3:** **Multivariate logistic regression of TBIC Practice using predictor factors identified in univariate logistic regression**

**Characteristics**	**Poor Practice**	**Good Practice**	**COR (95% CI)**	**AOR (95% CI)**
Job location				
Laboratory and Pharmacy	23	53	2.304 (1.255, 4.233)*	1.38 (0.674,2.829)
Wards	33	86	2.606 (1.519,4.471)*	0.509 (0.257, 1.008)
OPD	59	59	1	1
TBIC Knowledge				
Yes	54	179	10.64 (5.852, 19.35)*	10.667 (5.77,19.72)*
No	61	19	1	1

Note:*Significant association.

## Discussion

A majority of the respondents were found to score “good” on TBIC knowledge and practice. In addition to the good overall knowledge about TBIC, the respondents also demonstrated good knowledge about the need for an infection prevention (IP) committee to implement TBIC activities, window opening, patient isolation, and patient education. Furthermore, most of the respondents saw the need to prioritize TB patients to minimize hospital stay and the need for healthcare worker screening to quickly detect TB infection. These positive findings may be attributable to the dissemination of TBIC guidelines, trainings, and supportive supervisions by the national and regional TB control programs as well as the support from nongovernmental organizations working on TBIC.

The lower knowledge level regarding respirators and the use of fans may be attributable to unavailability of this equipment in the respective health facilities. Those who use respirators and fans probably received them from organizations working in partnership with the government on TBIC.

The proportion of TBIC knowledge questions answered correctly for all participating health professionals was 70%. On this measure, health workers’ knowledge was higher than that reported from the United States (median 55%) [13]. Eighty-six percent of the respondents in our study knew that educating patients on cough etiquette is important. This finding is in line with a report from the United States [14]. Knowledge levels in our study that are similar to or higher than those reported by healthcare workers in the United States could be explained by the high TB patient load in the study hospitals: The frequent exposure respondents in our study have to TB cases provides frequent opportunities to learn about TB treatment and prevention.

Of the health professionals in our study, 74.4% scored “good” knowledge in TBIC. This was better than rates reported from Russia (“overall scores were low”) [15] and the Philippines (misunderstanding of TB infection transmission despite high magnitude of TB in the general population) [16]. On the other hand, proportions of healthcare workers with “good” knowledge from Iraq (95.5%) [17], Saudi Arabia (81.8%), and Bangkok (85%) are higher than ours [18,19]. Explaining this knowledge difference is generally difficult; however, variations in the rates of “good” knowledge across countries could be attributed to the different settings and methodologies used by different studies.

TBIC knowledge was not found in our study to have any association with years of service, unlike the findings from Iraq [17]. Only 18.8% of the respondents were found trained on TBIC. This is contradictory to the recommendation on giving training on TBIC before the initial assignment of each health worker to any unit in the health facilities [8].

Training was found to have a statistically significant association with TBIC knowledge--OR 3.631 and 95% CI (1.496, 8.813). This was not the case in studies from the United States (on medical residents) [13] and Australia (on new graduate nurses) [20]. We found no significant differences in knowledge by category of healthcare workers, whereas in a study of Russian health workers physicians scored significantly higher than nurses and laboratory staff [15]. In our study a higher proportion of the respondents (91.4%) trained on TBIC responded as having contact with TB patients/suspected cases, while a study in South Africa found the opposite [21].

Our findings also revealed that only 34.2% of the healthcare professionals felt that a respirator should be worn to prevent TB infection transmission; this is far below what is reported from the United States (65% of those with no TB patient contact and 88% of those with patient contact) [14]. The unavailability of respirators in our context is the most likely reason for the reportedly low levels of this practice.

While training on TBIC was significantly associated with window opening and getting tested for TB when exposed to TB, it was not found to be a predictor of the other specific practice questions or of overall TBIC practice. This finding is contradictory to the report from Australia [20]. The lack of association between training and practice may be due to an emphasis on the theoretical aspects of training rather than skill-based components. Similarly, though we found statistically significant differences in window opening practices between those trained on TBIC and those not, OR 1.9 and 95% CI (1.02, 3.75), there were no significant differences in other standard IP practices (educating patients on cough etiquette, giving priority to suspected cases of TB) by training. This indicates that the basic minimum practice for healthcare facilities caring for patients suspected of air born diseases (including TB) is not in line with recommendations [8,22]. The Ethiopian TBIC guidelines clearly state that healthcare providers should triage and fast-track TB suspected cases, educate on cough etiquette, improve the cross ventilation of the room by opening windows, adjust seating arrangements, isolate TB suspected cases in the waiting area and wards, provide ambulatory management, conduct routine TB screening, follow up on TBIC activity implementation by the IP committee, and use respirators and fans to minimize TB infection [8].

Overall, 63.3% of the respondents scored “good” practice, where the median percent of TBIC practice questions correctly answered was 50%. This is much better than what is reported from Iraq (38.2%) [17]. However, a study from Bangkok revealed that all of the IP committee members and 85% of the hospital personnel attempted to implement TBIC measures [18].

Concerning specific practices, 21.8%, 32.3%, 44.2%, 40.8%, 30.2%, and 39.4% of the health professionals working in the outpatient department (OPD), TB/HIV clinic, medical ward, other wards, laboratory, and pharmacy, respectively, prioritize TB patients. This is much lower than what is observed from Bangkok [18]. Mask usage is reported by only 21.1% of the respondents, which is much lower than that reported from Bangkok [18] and South Africa [21]. The difference in these findings may be attributed to the unavailability of the masks. Window opening is practiced by the majority (64.9%) of respondents; this is much better than findings from the Philippines (39%) [23]. Nurses, pharmacy health professionals, and laboratory professionals are found to be better than physicians and health officers in giving priority for TB patients; however, it is not statistically significant, unlike the finding in Saudi Arabia [19].

In contrast to the high scores under each knowledge question, a majority of the health professionals did not demonstrate appropriate TBIC practice; only 21% use masks, 33.5% prioritize TB patients, and 39.9% use a fan (ventilator). The discrepancy between healthcare workers’ knowledge on the one hand and practice on the other is probably attributed to shortage or unavailability of supplies like fans (ventilators) and respirators (masks). Hence, health care workers have not had opportunities to put their knowledge into practice in an effective way.

Working in the wards, pharmacy, and laboratory were significant predictors of good practice compared to working in the outpatient department. This could probably be due to exposure of suspected or confirmed cases of TB patients coming to each of these units. TB suspects or cases come to these units for inpatient management, to get a sputum examination done, and to collect drugs for respiratory tract infections with documents indicating TB. These documents (patient chart, sputum laboratory request paper, and prescription papers) bring the issue of TB infection transmission to the attention of the healthcare workers in these units and alert them to be vigilant in IP. On the other hand, patients come to the outpatient clinics with numerous complaints, and only after a full clinical evaluation will suspicion of TB come to mind. By then, it is already too late to implement good TBIC measures.

The findings of this research were in line with what one may expect under each TBIC practice question: Health professionals with good TBIC knowledge were more likely to implement the TBIC practices compared to those with poor knowledge, OR 10.6 and 95% CI (5.8, 19.3).

In this study, though univariate regression showed mulitple predictor variables, AOR revealed that training is the only statistically significant determinant of TBIC knowledge. Similarly, multivariate logistic regression revealed that good TBIC knowledge is a strong determinant for good TBIC practice.

### Limitations of the study

There is limitated reference material available to compare our study with other studies done in similar settings.

Observing practices may produce more accurate results than asking about practices in a questionnaire, but this study did not include observation as a data collection method.

## Conclusions

A majority of the respondents were found to have good TBIC knowledge and practice. Training on TBIC appears to be a strong determinant of knowledge of TBIC, while knowledge of TBIC was a strong predictor of good TBIC practice.

On the other hand, training on TBIC on its own did not appear to have any positive influence on TBIC practice. This could be due to emphasis given to the theoretical aspects of training rather than skill-based components. Training healthcare professionals with emphasis on skills rather than theory is vital to strengthening the implementation of TBIC activities. The relatively low level of knowledge regarding respirators and fans/ventilators is probably due to the unavailability of these supplies, hence the availability of these supplies can improve their utilization and TBIC practice overall. Further research to investigate the reasons why training is not associated with better practice is recommended (Additional file 1).

## Additional file

Additional file 1
**Structured Questionnaire with consent form for the Assessment of TB Infection Control knowledge and Practice by Health Care Workers in North West Ethiopia; 2011.**
