# Supplementary material for: Knowledge and practice of tuberculosis infection control among health professionals in Northwest Ethiopia; 2011
Source: BMC Health Serv Res. 2014 Nov 19;14:593. doi: 10.1186/s12913-014-0593-2 (PMC4240849; doi:10.1186/s12913-014-0593-2)
Supplement: Additional file 1 — Structured Questionnaire with consent form for the Assessment of TB Infection Control knowledge and Practice by Health Care Workers in North West Ethiopia; 2011. [file 12913_2014_593_MOESM1_ESM.doc]

# Additional file 1: Structured Questionnaire with consent form for the Assessment of TB Infection Control knowledge and Practice by Health Care Workers in North West Ethiopia; 2011

**Oral Consent form፡**

**Introduction of study staff/Data collector**

Hello! My name is __________________________. My team and I are conducting tuberculosis infection control knowledge and practice assessment in North West Ethiopia.

**Brief description of the study**

The assessment is designed to answer the following key questions. What proportions of health professionals have tuberculosis infection control knowledge? What proportion of health professionals are implementing tuberculosis infection control measures? What are the factors associated with TBIC knowledge and practice?

If you agree to participate, here is what will happen. You will be asked to answer some questions about your sociodemographic characteristics, questions on your TBIC knowledge and practice. The results are important to inform future TBIC policy. Information from similar facilities in this region will be collected and analyzed together. There will be no Information reported that could identify the facility. No maps will be made that could be used to identify individual respondents or facilities by name or location. Your participation is voluntary but greatly appreciated. There will be no negative consequences to you if you decide not to participate.

Do you have any questions to ask me about this assessment?

If you have further questions or worries about the study, you are free to contact the principal investigator of the study (Chanie Temesgen Bayieh) in Bahir Dar, Ethiopia (chanie.tem@gmail.com).

We would like to ask you, if you are willing to participate.

Oral consent: Yes or No (circle appropriate answer)

Location code: __________________

Date: ___ / ___ / ____ (MM/DD/YY)

**Section 1. Socio-demographic and other General Characteristics**

| **Ser No** | **Question** | **Response** | **Response/Code** | | **Skip to Q** |
| --- | --- | --- | --- | --- | --- |
| 101 | Age | ( ) Years | | |  |
| 102 | Sex | M  F | 1  2 | |  |
| 103 | Educational Background | Diploma  First Degree  2^nd^ Degree and above | 1  2  3 | |  |
| 104 | Profession | Physician  Health Officer  Nurse/Midwife  Laboratory HW  Pharmacist HW  Others. Specify( ) | 1  2  3  4  5  6 | |  |
| 105 | Current Assignment Place (Job location) | OPD  TB / HIV Care units  General Medical Ward patients  Other wards    Laboratory  Pharmacy  Other. Specify ( ) | 1  2  3  4  5  6  7 | |  |
| 106 | Number of Months/Years of service after the last graduation | ( ) Month(s) /year(s) | | | Underline Mos./Yrs |
| 107 | Have you ever taken training on TB IC? | Yes  No | | 1  2 | 201 if 2 |
| 108 | When was the training conducted? | In the past 1 year  In the past 2 years  In the past 3/more years | | 1  2  3 |  |
| 109 | How long was the duration of the training? | One day or less  2-3 days  more than 3 days | | 1  2  3 |  |
| 110 | How many TB suspects or confirmed cases do you encounter /provide service in one day? | ( _________ ) patients | | 1 |  |
|  |  | Don’t know | | 2 |  |

**Section 2. Knowledge on Tuberculosis Infection Control (TB IC)**

| **Ser No** | **Question** | **Response** | | | **Code** | **Skip to** |
| --- | --- | --- | --- | --- | --- | --- |
|  |  | **Yes** | **No** | **Don’t Know** |  |  |
| 201 | The door and window of a room should be left open whenever a patient suspected or confirmed to have TB is in the room. | 1 | 2 | 3 |  |  |
| 202 | Patients suspected or confirmed to have TB should be kept separately from the rest of the patients | 1 | 2 | 3 |  |  |
| 203 | HCWs should try to minimize the time TB patients spend in the health facility | 1 | 2 | 3 |  |  |
| 204 | Surgical masks can’t protect the HCW from inhaling M. tuberculosis containing aerosols | 1 | 2 | 3 |  |  |
| 205 | Respirators can protect the HCW from inhaling M. tuberculosis containing aerosols | 1 | 2 | 3 |  |  |
| 206 | TB patients have to be educated to cover their mouth with a handkerchief or scarf | 1 | 2 | 3 |  |  |
| 207 | Every health facility should establish an IC committee | 1 | 2 | 3 |  |  |
| 208 | Patients suspected or confirmed to have TB and are coughing should get priority to be seen by a nurse/doctor first | 1 | 2 | 3 |  |  |
| 209 | Regular screening of HCWs for presence of TB is one of the TB infection control measures | 1 | 2 | 3 |  |  |
| 210 | Fans (Ventilators) can be used in TB wards to reduce the transmission of TB in TB wards | 1 | 2 | 3 |  |  |

Section 3. Practice of Standard TB Infection Control Measures

| **Ser No** | **Question** | **Response** | | | **Code** | **Skip to** |
| --- | --- | --- | --- | --- | --- | --- |
|  |  | **Always** | **Sometimes** | **Never** |  |  |
| 301 | Do you open the window whenever a patient suspected or confirmed to have TB is in the room? | 1 | 2 | 3 |  |  |
| 302 | Do you use a mask/respirator whenever you are treating TB patients/suspects? | 1 | 2 | 3 |  |  |
| 303 | Do you try to see coughing patients at first, in other words, if there are coughing patients in the waiting area, do you give them priority? | 1 | 2 | 3 |  |  |
| 304 | Do you educate your TB patients on cough etiquette? (That is covering of mouth while coughing, not spitting on the floor, etc…) | 1 | 2 | 3 |  |  |
| 305 | If you were supplied with fans, would you turn them on while you are treating TB suspects or confirmed cases? | 1 | 2 | 3 |  |  |
| 306 | Having been in contact with TB patients, would you test for TB in case you have cough? | 1 | 2 | 3 |  |  |
